# Feasibility study of a LED light irradiation device for the treatment of chronic neck with shoulder muscle pain/stiffness

**DOI:** 10.1371/journal.pone.0276320

**Published:** 2022-10-17

**Authors:** Keiichi Odagiri, Katsuya Yamauchi, Masahiro Toda, Ayako Uchida, Hiromi Tsubota, Kazuyoshi Zenba, Hiroaki Okawai, Hideo Eda, Seiichiro Mizuno, Hiroaki Yokota

**Affiliations:** 1 Center for Clinical Research, Hamamatsu University Hospital, Hamamatsu, Japan; 2 Department of Rehabilitation Medicine, Hamamatsu University School of Medicine, Hamamatsu, Japan; 3 Isehara Clinical Trial Center, Zenba-Acupuncture and Moxibustion Clinic, Isehara, Japan; 4 Kenkou Data House Inc., Sendai, Japan; 5 The Graduate School for the Creation of New Photonics Industries, Hamamatsu, Japan; Monash University, AUSTRALIA

## Abstract

Neck with shoulder muscle stiffness/pain is a common disorder. Commonly used physical therapy, pharmacotherapy, acupuncture, and moxibustion only temporarily alleviate the disorder in most cases, thus the disorder often recurs. Low power laser therapy is often used for neck and shoulder stiffness/pain and has been effective in clinical trials. In this study, we evaluated the safety and effectiveness of a newly developed self-care device for disorders including neck with shoulder muscle stiffness/pain. The device incorporates light-emitting diodes (LEDs), which are safer than lasers, as its light source. Ten adults with neck with shoulder muscle stiffness/pain were subject to LED irradiation (wavelength 780 nm ± 15 nm, output 750 mW, power density 3.8 W/cm^2^, energy density 5.7×10^2^ J/cm^2^) for 3 minutes on the affected shoulder at a standard acupuncture point (GB21, Jianjing). Immediately after irradiation, the subjective symptoms of the neck with shoulder muscle stiffness and pain evaluated by a visual analog scale were improved from 58.3 mm ± 18.7 mm to 45.5 mm ± 21.5 mm and from 45.8 mm ± 23.3 mm to 39.4 mm ± 21.8 mm, respectively. The symptoms further improved after 15 minutes of irradiation. The skin temperature at the irradiated point increased from 34.3°C ± 1.1°C to 41.0°C ± 0.7°C. The increase in skin temperature was observed within approximately 5 cm of the irradiated area. There was no effect on the heart rate variability, a measure of the autonomic nervous system; however, the baroreflex sensitivity was slightly increased. No irradiation-related adverse skin events were observed. Our LED irradiation device was found to be safe, and it improved the subjective symptoms of muscle stiff neck with shoulders.

## Introduction

Chronic neck with shoulder muscle pain/stiffness is a common symptom that can limit an individual’s daily life [1]. The 12-month prevalence of neck pain in the world population, and Japanese male and female adults is 30%–50%, 5.7%, and 11.4%, respectively [2, 3]. Although several treatments, such as pharmacotherapy, physical therapy, exercise, acupuncture, and moxibustion are available to relieve symptoms, individuals often have recurrent episodes [4–8]. As a result, the economic impact is increasing, and the development of evidence-based, cost-effective treatments is needed [9].

Low-level laser therapy (LLLT) is a medical device frequently used to relieve pain and stimulate acupuncture points in clinical settings. The safety and efficacy of LLLT for chronic neck pain were shown in several clinical trials [10–12]. The Japanese government recently promoted the development of self-care devices for chronic disease, and various low-level laser devices are available as self-health care equipment for chronic neck with shoulder muscle pain/stiffness; however, most of the devices have no medical device certification and no safety and efficacy information. Thus, in this feasibility study, we aimed to assess the safety and efficacy of our newly developed light-emitting diode (LED) light irradiation device for chronic neck with shoulder muscle pain/stiffness treatment.

## Materials and methods

### Ethics statement

The study was conducted according to the guidelines of the Declaration of Helsinki and the Clinical Trials Act. The clinical research review board of the Hamamatsu University School of Medicine (1–20–1 Handayama, Higashi-Ku, Hamamatsu, Shizuoka, Japan) approved the study in January 2021 (approval number: C018-2020). Written informed consent was obtained from all patients before inclusion in the trial. This trial was registered at the Japan Registry of Clinical Trials (jRCTs042200092).

### Study participants

Participants were recruited from January 2021 to March 2021 via open public recruiting. Eligible participants were adults with muscle stiff necks with shoulders. The exclusion criteria were participants with cervical spine disease (e.g., whiplash injury, disc herniation, facet arthropathy), those receiving medical treatment for a stiff neck and shoulder provided by a physician, those using a poultice bandage or receiving medical therapy, acupuncture, moxibustion, or massage, those with a skin disorder at the treatment site.

### Study design and protocol treatment

A single-center, single-arm, open-label feasibility study was conducted at Hamamatsu University Hospital Translational Research Unit, Hamamatsu, Japan from February 2021 to March 2021. Participants received a single irradiation of high-intensity infrared LED light (wavelength 780 nm ± 15 nm, output 750 mW, power density 3.8 W/cm^2^, energy density 5.7×10^2^ J/cm^2^) (Prototype model; Saciperere Japan, Numazu, Japan) six times at three-second intervals for 30 seconds. The LED light was directed to the affected shoulder at a standard acupuncture point (Jianjing, GB21 of the World Health Organization’s standard acupuncture nomenclature, middle of shoulder foot gallbladder meridian,) for a total of 3 minutes while the patient was in a seated position. Jianjing was chosen by an experienced acupuncture and moxibustion practitioner.

### Efficacy measurements

Primary outcome measures were differences in the severity of the neck with shoulder muscle stiffness and pain as quantified with a 100 visual analog scale (VAS) ranging from 0 (no neck and shoulder stiffness or pain) to 100 (the most severe neck and shoulder stiffness or pain imaginable) before and after treatment. Secondary outcome measures included differences in skin temperature, heart rate variability, and baroreceptor reflex sensitivity (BRS). The skin temperature was measured by standard thermography. The heart rate variability and BRS were measured by a noninvasive hemodynamic and electrocardiogram monitor (Finapres NOVA; Zero C Seven, Inc., Tokyo, Japan). The pressure pain threshold measured by an algesiometer (TDM-N1, Sato Shoji Inc. Kawasaki, Japan) was also selected as a secondary outcome measure; however, it could not be measured because of the wrong usage of the algesiometer. The VAS and skin temperature were assessed immediately before, 0, and 15 minutes after treatment. The BRS and heart rate variability were continuously measured 5 minutes before to 15 minutes after treatment. All measurements were performed when the subject was in a seated position.

### Safety assessments

The safety of the subjects was measured by recording the sitting systolic and diastolic blood pressure and pulse rates immediately before and 15 minutes after treatment. Adverse effects were also reported 0–7 days after treatment.

#### Exploratory measurements

The differences in the surface electromyogram and an ultrasonography under weight load (a weight lifting task of 2–3 kg for 1.5–2 minutes) with the trapezius muscles before and after treatment were also assessed (detailed procedures in S1 File).

#### Sample size

A sample size calculation was not performed because this was a feasibility study. Ten patients were the minimum sample size to show the effectiveness of the LED light device.

#### Statistical analysis

The full analysis set for all patients who received study treatment was the primary analysis set for all planned efficacy and safety analyses. Values are expressed as the mean ± standard deviation (SD) of the indicated numbers or proportions (%). Before computing the statistical analysis, the distribution of all measurements, except for heart rate variability, was verified to follow a normal distribution using Q-Q plots. The parameters of heart rate variability were confirmed to follow a normal distribution by natural log transformation. The VAS scores and skin temperature immediately before and at 0 and 15 minutes and the mean of 5-minute periods (−5 to 0 minutes before treatment, 0 to 5 minutes after treatment, and 10–15 minutes after treatment) of the natural log-transformed heart rate variability (lnHF, lnLF, and ln[LF/HF]) were compared using the General Linear Model (GLM) [13]. The mean of 5-minute periods of BRS, and the diastolic and systolic blood pressure were compared using age-adjusted GLM. The pulse rate was compared using GLM. *P* values less than 0.05 or 0.025 (if multiple comparisons) (2-tailed) were considered statistically significant. Statistical analysis was performed using IBM SPSS Statistics version 25.0 (IBM Corporation, Armonk, NY, USA).

## Results

### Study participant characteristics

**Fig 1:**
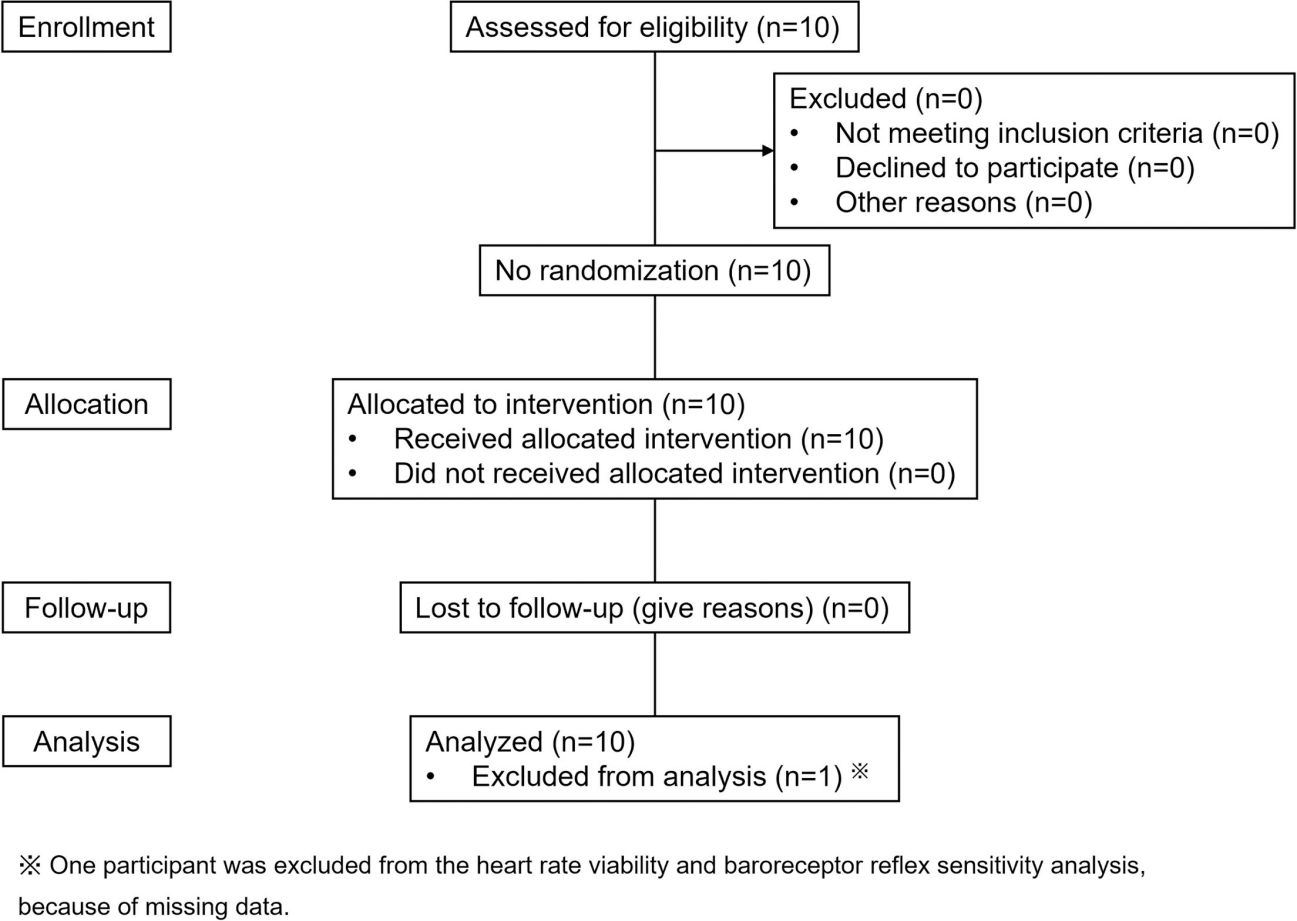
CONSORT diagram.

A total of 10 participants (2 males and 8 females, age 43.7 ± 10.3 years (range 29–62 years old)) participated in the study between February 2021 and March 2021. Seven of ten participants received treatment on the left shoulder, and three received treatment on the right shoulder. No participant received LED light irradiation on both shoulders. All participants completed the study and were analyzed; one participant’s heart rate viability and BRS were not recorded due to a malfunction of the Finapres NOVA (Fig 1).

### Efficacy

**Fig 2:**
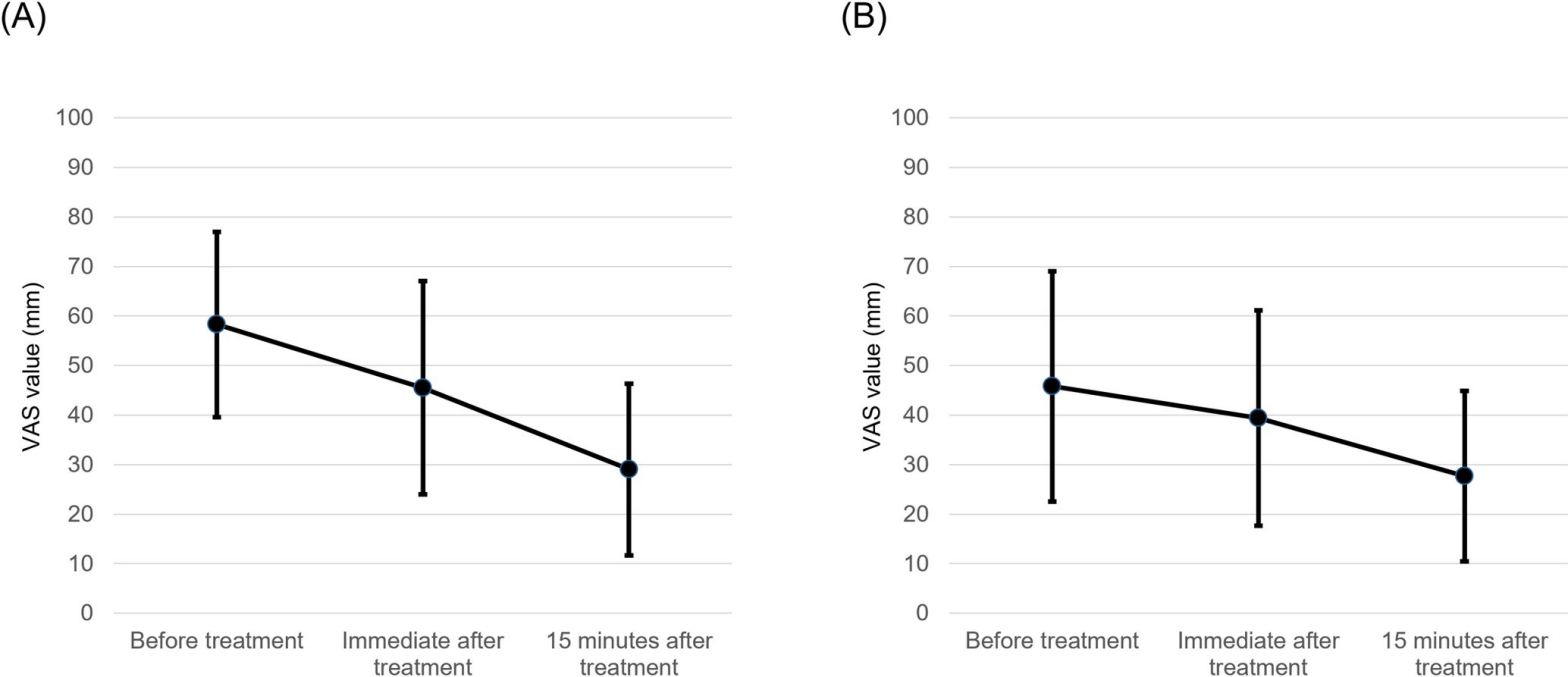
The effect of LED light irradiation on the VAS for chronic neck with shoulder muscle stiffness and pain for each subject. Variations in VAS scores of the (A) neck with shoulder muscle stiffness and (B) pain before, immediately after, and 15 minutes after treatment.

The stiffness intensity was significantly decrease (*p* = 0.018) immediately after treatment (VAS score: 45.5 mm ± 21.5 mm) than before treatment (VAS score: 58.3 mm ± 18.7 mm). This improvement was sustained for 15 minutes after treatment (VAS score: 29.0 mm ± 17.3 mm, *p* = 0.002, compared with before treatment) (Fig 2A). The pain intensity tended to decrease both immediately after treatment (VAS score: 39.4 mm ± 21.8 mm, *p* = 0.142) and 15 minutes after treatment (VAS score: 27.7 mm ± 17.2 mm, *p* = 0.028) compared with before treatment (VAS score: 45.8 mm ± 23.3 mm); however, these pain intensity reductions did not reach statistical significance. (Fig 2B).

**Fig 3:**
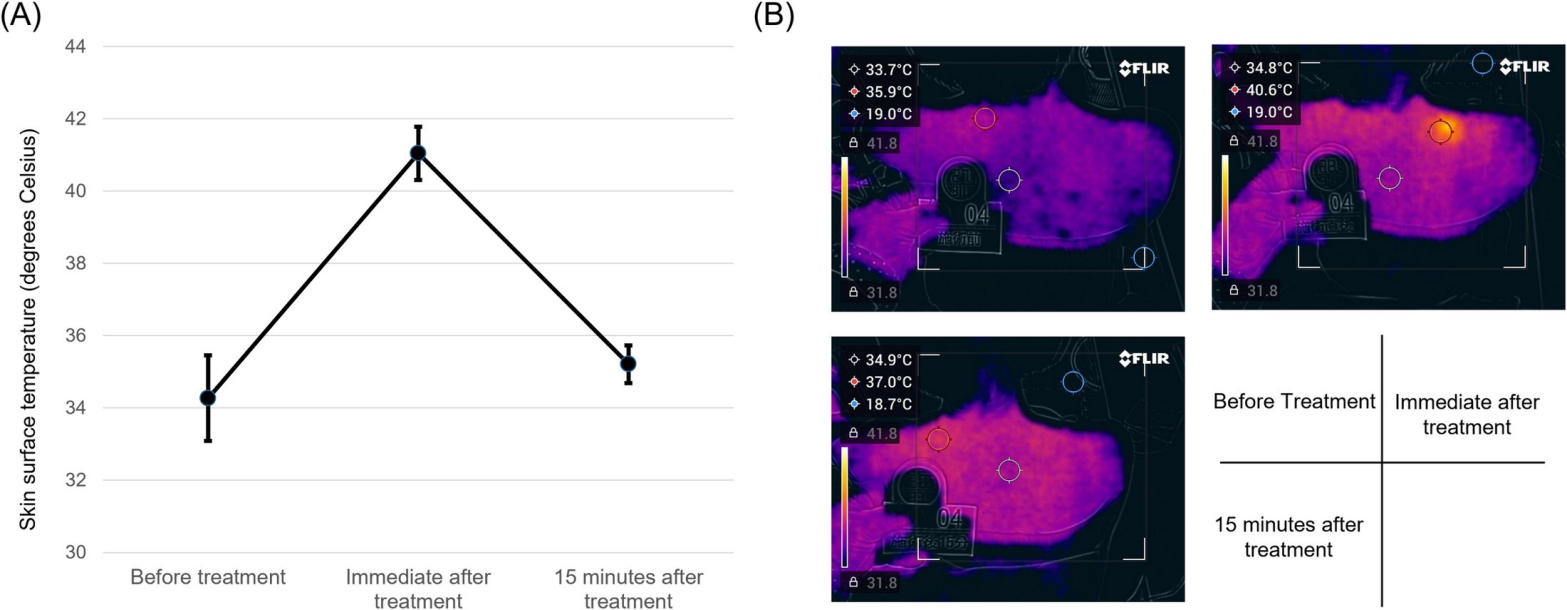
The effect of LED light irradiation on the skin surface temperature. (A) Variations in the skin surface temperature around the LED light irradiation site of each study participant before, immediately after, and 15 minutes after treatment. (B) Thermography images of the skin surface before (upper left), immediately after (upper right), and 15 minutes after LED irradiation (lower left).

The skin surface temperature significantly increased 6.8°C ± 1.5°C at the acupuncture point from 34.3°C ± 1.1°C to 41.0°C ± 0.7°C with treatment (*p* < 0.001), and returned to the baseline temperature in 15 minutes (35.2°C ± 0.5°C, *p* = 0.033) (Fig 3A). Representative images are shown in Fig 3B.

An increase in skin surface temperature was observed within approximately 5 cm of the center of the irradiated area. Skin redness at the irradiation area caused by the skin temperature increase was observed in nine participants during the treatment or immediately after the treatment; however, the temperature decreased in six of the nine participants 15 minutes after treatment. No participant showed additional skin redness 7 days after the treatment.

**Fig 4:**
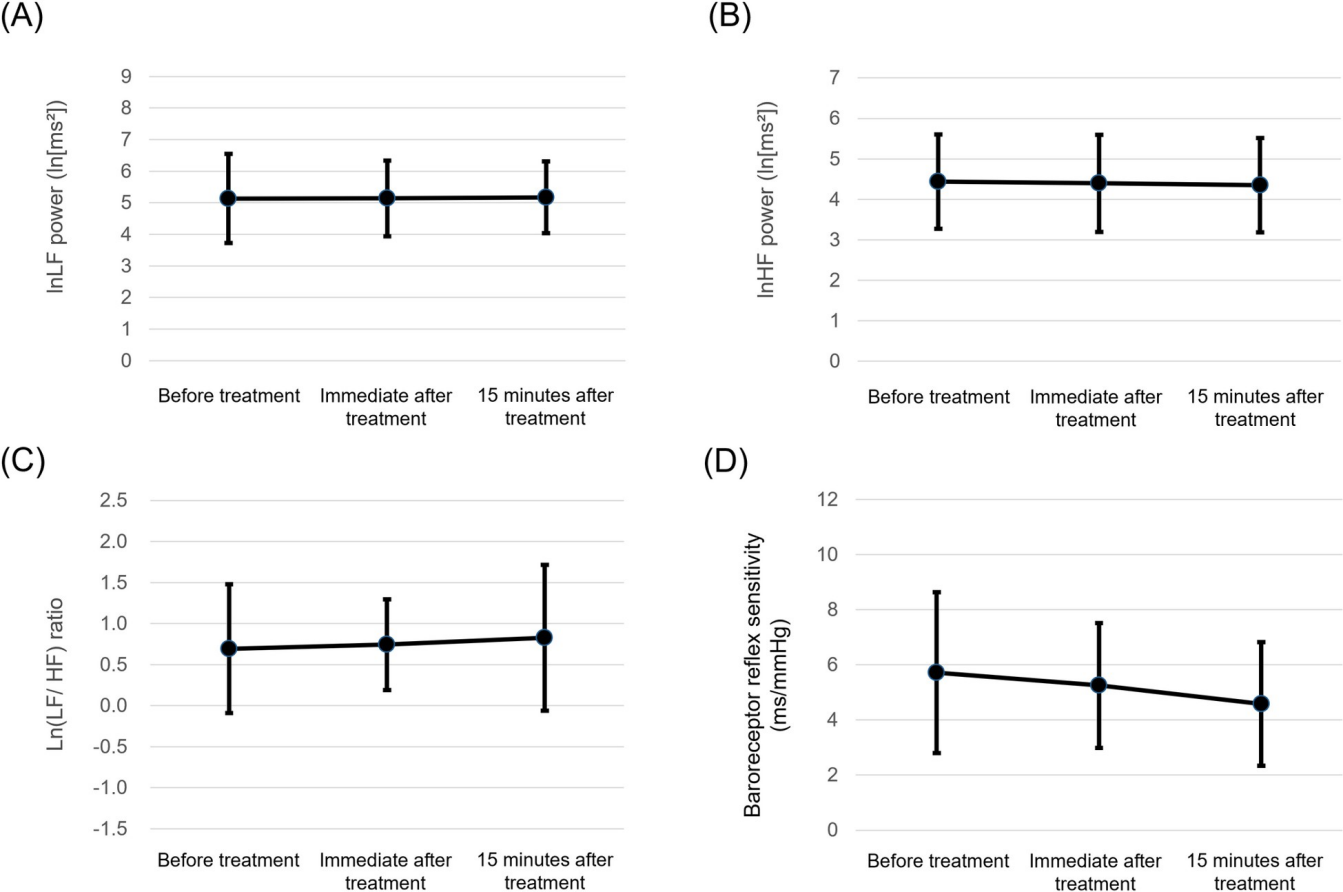
The effect of LED light irradiation on the heart rate viability and baroreceptor reflex sensitivity of each study participant. Variations in (A) low frequency power, (B) high frequency power, (C) low frequency power/high frequency power ratio, and (D) baroreceptor reflex sensitivity before, immediately after, and 15 minutes after LED light irradiation.

Fig 4A–4C shows the changes in low frequency power (0.07–0.15 Hz; LF), high frequency power (>0.15 Hz; HF), and LF/HF ratio indices of the heart rate viability. A treatment effect was not observed with the LF power, HF power, or LF/HF ratio. Furthermore, LED light irradiation did not show any effects on BRS (Fig 4D). Detailed variables are shown in Table 1.

**Table 1:** Changes in the heart rate variability and baroreceptor reflex sensitivity.

	Before treatment	Immediately after treatment	15 minutes after treatment	P value *	P value^†^
ln LF power (ln[ms^2^])	5.13 ± 1.41	5.14 ± 1.20	5.17 ± 1.14	0.976	0.861
ln HF power (ln[ms^2^])	4.44 ± 1.16	4.40 ± 1.20	4.35 ± 1.17	0.751	0.927
ln (LF/ HF)	0.69 ± 0.78	0.74 ± 0.55	0.82 ± 0.89	0.691	0.604
BRS (ms /mmHg)	5.72 ± 2.90	5.25 ± 2.30	4.58 ± 2.20	0.061	0.063

Data are expressed as mean ± SD. *Comparison between patients before and immediately after treatment. ^†^Comparison of the values before and 15 minutes after treatment. The General Linear Model was used to analyze the difference. LF, low frequency; HF, high frequency; BRS, baroreceptor reflex sensitivity.

### Safety

The systolic and diastolic blood pressures and pulse rates were comparable before and 15 minutes after treatment (the systolic blood pressures were 121.2 mmHg ± 11.1 mmHg (before treatment) and 120.8 mmHg ± 15.5 mmHg (15 minutes after treatment), *p* = 0.150; the diastolic blood pressures were 75.0 mmHg ± 6.8 mmHg (before treatment) and 72.4 mmHg ± 7.3 mmHg (15 minutes after treatment), *p* = 0.253; the pulse rates were: 75.8 ± 10.4 beats/minute and 72.0 ± 10.5 beats/minute, *p* = 0.149, respectively). No treatment-related advised effects were observed.

### Exploratory measurements

The surface electromyogram and echography underweight load with the trapezius muscles did not show any treatment-specific findings that indicate treatment mechanisms.

## Discussion

The present feasibility study showed the safety and efficacy of the LED-light irradiation device for chronic neck with shoulder muscle pain/stiffness treatment in Japanese subjects. In this study, 3 minutes of infrared LED light irradiation on Jianjing immediately relieved both subjective pain (changes in VAS score: −17.4%) and stiffness (changes in VAS score: −24.3%), followed by further improvement in relief after 15 minutes (changes in VAS score: pain, −37.5%, stiffness, −48.3%). The skin surface temperature significantly increased 6.8°C ± 1.5°C at the irradiation site. No adverse effects, such as thermal burn at the treatment site associated with LED irradiation, were observed even 7 days after the irradiation. These results were consistent with previous systematic review and meta-analysis [14, 15]. The effect size on pain relief assessed by VAS shown in the results of this study was a reduction of 18.1 mm, suggesting that this may be equivalent to the effect size of 19.7 mm shown in previous systematic reviews [15].

To the best of our knowledge, there is no evidence of pain treatment by LED irradiation. Thus, we referred to the evidence of pain treatment using LLLT to determine the irradiation parameters. LLLT has been applied to the treatment of chronic or acute pain, with accumulating evidence of safety and efficacy. As a result, some reports have shown that LLLT can relieve rheumatoid arthritis pain, morning stiffness, and chronic and acute back pain with few side effects [16–18]. However, the efficacy of LLLT varies from disease to disease. The effectiveness of LLLT in the treatment of carpal tunnel syndrome has not been established [19]. Thus, the effects of LLLT on pain varies across diseases. Disease-specific heterogeneity in LLLT efficacy is thought to depend on disease-specific characteristics and several factors, including laser wavelength and power, and the irradiation time of the treatment site. However, the appropriate irradiation dose, treatment site, and technique for each disease has not been established. A previous systematic review reported that LLLT at infrared wavelengths of 780 nm, 810 nm, and 904 nm showed efficacy in patients with neck pain [14]. Another systematic review indicated that 820–830 nm doses in the range of 0.8–9.0 J per point with an irradiation time of 15–180 seconds are safe and effective for the management of neck pain. Furthermore, one clinical trial showed that a high dose (54 J) of 838 nm LLLT did not have any adverse effects [19]. Considering the wavelengths, irradiation time, and output parameters that have been reported to be safe and effective with LLLT, we chose a wavelength of 780 nm ± 15 nm, an output of 750 mW, 180 seconds (i.e., 135 J) of irradiation, and selected Jianjing as the treatment site, which is known as a trigger point in stiff shoulders. Our treatment device was safe to use, and reduced the subjective symptoms of neck and shoulder stiffness. Although the safety of LLLT has been proven in previous studies [15, 19–21], this LED irradiation treatment device may be safer than LLLT and thus likely to be competitive in healthcare markets. Indeed, LEDs emit broadband light that is not monochromatic, is less directional, and is noncoherent compared with lasers.

In this study, we showed that our LED irradiation device can improve subjective symptoms of chronic neck with shoulder muscle pain/stiffness; however, the detailed mechanism has not been clarified. Several mechanisms of pain relief via infrared irradiation have been proposed. Possible mechanisms include anti-inflammatory effects via reduction of inflammatory cytokines, oxidative stress reduction, and skeletal muscle fatigue [22, 23]. Studies in rodents have shown that infrared laser irradiation selectively suppresses Aδ and C fibers [24, 25]. Furthermore, inhibition of neuromuscular junction transmission has also been proposed as a mechanism of infrared laser on myofascial pain and trigger points [26, 27]. Thus, infrared laser-mediated nerve inhibition is considered to be one of the most powerful mechanisms for pain relief. Another possible mechanism for pain relief from infrared irradiation is the increase in body temperature at and near the irradiated site. Local thermotherapy has been applied to the treatment of acute and chronic pain. Although there is no established evidence, moxibustion, a traditional Chinese heat therapy, is often used for pain relief. The effects of thermotherapy on physiological effects have been studied. Body surface heating increases the skin temperature, which in turn increases blood flow and metabolism. Additionally, an increase in skin temperature decreases the rigidity of the fascial tissue [28]. In previous animal studies, local thermotherapy was reported to suppress non-sensory signals in the spinal cord and brain stem via increased activity of small unmyelinated C-fibers [29]. It has also been reported that local thermotherapy decreases the activity of γ fibers and the stretch sensitivity of muscle spindles [30]. In the current study, we demonstrated that LED light irradiation increased the skin surface temperature by 6.8°C at the irradiation site, and the increase spread approximately 5 cm. One possible mechanism for the effectiveness of our device may be the physiological changes caused by the increase in skin temperature.

Our study has several limitations. First, the LED irradiation is a single application, and irradiation conditions are constant; as a result, the optimal number of applications, output, and irradiation time have not been evaluated. The optimal irradiation conditions should be established in further clinical studies by changing the number of consecutive applications and irradiation parameters. Second, selection of study participants via open recruitment could cause selection bias, such as a healthy worker effect, and age and gender imbalance; however, the LED light irradiation device used in this study is intended to be developed as a self-care device in the future, and the results obtained in this study could serve as basic data for future large-scale clinical studies to create evidence for the LED light irradiation devices. Third, our study had a small sample size; however, it was designed to determine whether it would be feasible to conduct the next clinical study, and we believe we achieved this purpose. Based on the results of this study, a large-scale clinical trial is planned in the future.

## Conclusion

A single irradiation with LED lights (wavelength 780 nm ± 15 nm, power 750 mW, power density 3.8 W/cm^2^, energy density 5.7×10^2^ J/cm^2^, duration 3 minutes) was safe to use in the treatment of chronic neck with shoulder muscle pain/stiffness, relieving patients’ pain and stiffness. Further studies are needed to determine the optimal irradiation conditions and to elucidate the mechanism of action.

## Supporting information

S1 Checklist TREND statement checklist. (PDF)

S1 File Detailed implementation procedures for exploratory measurements. (DOCX)

S1 Dataset (XLSX)

S1 Protocol (DOCX)

S2 Protocol (DOCX)

S3 Protocol (DOCX)
