# Supplementary material for: Feasibility study of a LED light irradiation device for the treatment of chronic neck with shoulder muscle pain/stiffness
Source: PLoS One. 2022 Oct 17;17(10):e0276320. doi: 10.1371/journal.pone.0276320 (PMC9576044; doi:10.1371/journal.pone.0276320)
Supplement: S1 File — (DOCX) [file pone.0276320.s002.docx]

The detailed procedure of surface electromyogram and ultrasonography under weight load (a weight lifting task of 2–3 kg for 1.5–2 minutes) with the trapezius muscles before and after treatment

**Surface electromyography (EMG)**

Perform a weight-loading test on the shoulder on the treatment side and evaluated muscle fatigue associated with the load with an EMG.

(1) The examination was performed in a sitting posture.

(2) The measurement sites were the trapezius muscle (especially from the neck to the shoulder) and the deltoid muscle at the treatment side.

(3) Both arms were placed on a desk at rest, and then baseline EMG measurements were taken for 15 seconds.

(4) Next, a weight of 2-3 kg was held with the treating side arm and held in the following order: after placing the upper arm was held in a forward horizontal position, and then the forearm flexed 90 degrees vertically upward. followed by, extending the forearm with holding the weight for 2 minutes. The arm of the non-treated side was in a posture to support the upper body. EMG was measured over time during this period.

(4) The maximum loading time is 2 minutes, but if the participant is unable to tolerate the weight load and postural maintenance, a shortening of up to 90 seconds was acceptable.

**Muscle ultrasonography**

Muscle ultrasonography was also performed on the trapezius muscles (especially from the neck to the shoulders) and deltoid muscles at rest and during weight loading.

(1) Avoid EMG electrode positions when performing ultrasonography.

(2) A comparison of muscle echo intensity on ultrasound (luminance, muscle contraction, muscle thickness, etc.) of the muscles under weight-loading was performed.
