# Supplementary material for: Feasibility study of a LED light irradiation device for the treatment of chronic neck with shoulder muscle pain/stiffness
Source: PLoS One. 2022 Oct 17;17(10):e0276320. doi: 10.1371/journal.pone.0276320 (PMC9576044; doi:10.1371/journal.pone.0276320)
Supplement: S2 Protocol — (DOCX) [file pone.0276320.s005.docx]

Trial study protocol

**Feasibility study of LED-light acupuncture and moxibustion for stiff neck and shoulder**

**Short title: Feasibility study of LED-light acupuncture and moxibustion for stiff neck and shoulder**

Version 1.1

January 29, 2021

Revision History

| Number of Editions | Date of Completion | Date of Implementation |
| --- | --- | --- |
| Version 1.0 | December 17, 2020 | After publication in jRCT |
| Version 1.1 | January 29, 2021 | After publication in jRCT |

**1.** **Matters related to the implementation system for clinical trial**

＜Medical institution and principal investigator＞

| Implementing medical institution | organization | Hamamatsu University Hospital |
| --- | --- | --- |
|  | location | 1-20-1 Handayama, Higashi-ku, Hamamatsu City, Shizuoka Prefecture |
|  | TEL | +81-53-435-2006 |
| principal investigator | Name | Keiichi Odagiri |
|  | Affiliation | Center for clinical research, Hamamatsu University Hospital |
|  | position | Associate professor |
|  | TEL | +81-53-435-2006 |

＜Each person responsible for engaging in clinical research other than the principal investigator＞

［Data Management］

| Name | not applicable |
| --- | --- |
| Affiliation |  |
| position |  |
| TEL |  |

[Statistical Analysis]

| Name | not applicable |
| --- | --- |
| Affiliation |  |
| position |  |
| TEL |  |

［Monitoring］

| Name | Kei Hiruta |
| --- | --- |
| Affiliation | Center for clinical research, Hamamatsu University Hospital |
| position | Clinical research associate |
| TEL | +81-53-435-2006 |

［Audit］

| Name | not applicable |
| --- | --- |
| Affiliation |  |
| position |  |
| TEL |  |

［Project management］

| Name | not applicable |
| --- | --- |
| Affiliation |  |
| position |  |
| TEL |  |

［study management］

| Name | Hiromi Tsubota |
| --- | --- |
| Affiliation | Center for clinical research, Hamamatsu University Hospital |
| position | Project manager |
| TEL | +81-53-435-2008 |

［Persons overseeing the research other than the Principal Investigator/Principal Investigator］

| Name | Hiroaki Yokota |
| --- | --- |
| Affiliation | The Graduate School for the Creation of New Photonics Industries |
| position | Associate professor |
| TEL | +81-53-484-2501 |

［Medical and technical departments and institutions］

　[acupuncture and moxibustion practitioner]

| organization | Isehara Clinical trial center, Zenba-acupuncture and moxibustion clinic |
| --- | --- |
| name | Kazuyoshi Zenba |
| location | 4-637-1 Isehara, Isehara City, Kanagawa Prefecture |

　[Evaluator]

| organization | Kenkou Data House Inc. |
| --- | --- |
| name | Hiroaki Okawai |
| location | 3-21-11 Katsura, Izumi-ku, Sendai-shi, Miyagi |

[Evaluator]

| organization | The Graduate School for the Creation of New Photonics Industries |
| --- | --- |
| name | Hideo Eda |
| location | 1955-1, Kurematsu-cho, Nishi-ku, Hamamatsu City, Shizuoka Prefecture |

[Evaluator]

| organization | The Graduate School for the Creation of New Photonics Industries |
| --- | --- |
| name | Seiichiro Mizuno |
| location | 1955-1, Kurematsu-cho, Nishi-ku, Hamamatsu City, Shizuoka Prefecture |

［Evaluator］

| organization | Department of Rehabilitation Medicine, Hamamatsu University School of Medicine |
| --- | --- |
| name | Masahiro Toda |
| location | 1-20-1 Handayama, Higashi-ku, Hamamatsu City, Shizuoka Prefecture |

［Research equipment provider］

| organization | Saciperere Japan |
| --- | --- |
| location | 3-30-32, Sekimachi Minami, Nerima-ku, Tokyo |

［Contract manufacturer of research equipment］

| organization | TAK SYSTEM INITIATIVE CORPORATION |
| --- | --- |
| location | 1295-3 Kobayashi, Hamakita-ku, Hamamatsu City, Shizuoka Prefecture |

**2. Matters related to the background of the clinical trial (including matters related to the outline of the drug product)**

2-0. Outline

Clinical Question: There are many self-care devices for stiff shoulders on the market, but their efficacy and mechanism have not been sufficiently verified.

Patient: Persons over 20 years old who are aware of stiff shoulders.

Intervention: Perform optical acupuncture.

Comparison: None

Outcome: Relief of subjective symptoms of stiff shoulders and clarification of the mechanism

Research Question: Will a single treatment using an LED acupuncture device on adults with shoulder stiffness cause any change in pain threshold evaluated by tenderness meter, subjective symptoms (shoulder stiffness, pain) evaluated by VAS, autonomic nerve function evaluated by Finapres NOVA, and baroreceptor reflex sensitivity compared to before the treatment. The results will be compared with those before the treatment to see if any changes occur.

2-1. Background and Significance of Clinical trial

(a) Current status of healthcare products and positioning of this research and development

In the Health and Medical Care Strategy approved by the Cabinet on March 27, 2020, the promotion of the healthcare industry outside the public insurance is mentioned as a new industry creation that contributes to the formation of a healthy and long-lived society. (PD: Yoshiyuki Myonaka), and the development of healthcare devices has become one of the pillars of the national strategy in health promotion and industrial growth. Against this backdrop, Sasiperere Japan Ltd. has embarked on the development of self-care products targeting "stiff shoulders. It is known that a large number of people are aware of stiff shoulders (see below), and in addition to visiting a medical institution to treat the underlying disease, massage and acupuncture, and moxibustion are widely used to deal with this problem. Acupuncture and moxibustion are performed by acupuncturists who have specialized skills and knowledge and are nationally certified. Sasiperere Japan Ltd. has been developing an LED light acupuncture and moxibustion device to provide a painless, safe, compact, and easily usable device for general users by converting the specialized skills of acupuncturists into IOT.

Associate Professor Yokota of the Graduate University for the Creation of New Photonics Industries and Sasiperere Japan Ltd. has been selected by the Hamamatsu Photon Valley Center, Hamamatsu Regional Innovation Promotion Organization, to participate in the 6th A-SAP Industry-Academia-Government-Finance Collaborative Innovation Promotion Project for FY2020, for "obtaining evidence of the efficacy of LED optical acupuncture. A clinical trial was planned to clarify the actual clinical efficacy in humans using the LED optical acupuncture device. The purpose of this R&D project is to use a prototype LED optical acupuncture and moxibustion device for single-use on people who are aware of stiff shoulders and to examine whether or not the device improves symptoms, is safe, and affects physiological indices. In the future, using this research as a step, the company aims to develop a device that enables the identification of "acupuncture points" using AI in combination and launch it as a health care product.

(b) Status of target diseases in Japan and overseas (including epidemiological data on target diseases)

Shoulder stiffness is a subjective symptom, generally a syndrome "characterized by symptoms and complaints of discomfort, discomfort, and dull pain mainly caused by muscle tension from the lower occipital area to the posterior neck, back of the shoulder blade, and shoulder joint.^1)^ According to the 2019 National Survey of Living Conditions (Ministry of Health, Labour and Welfare, Office of Household Statistics, Ministry of Health, Labour and Welfare, July 17, 2020), 57.2/1000 males (2nd highest prevalence) and 113.8/1000 females (1st highest prevalence) were affected by the syndrome.^2)^

(Figure, Top 5 Symptoms of Prevalence by Gender, reproduced from the 2019 National Survey of Living Conditions)

Causes of stiff shoulders are classified as intrinsic (primary), symptomatic, and psychogenic. Intrinsic causes are those that have no underlying disease and are considered at risk from poor posture, muscle weakness due to lack of exercise, inappropriate exercise, overwork, cold, stress, and aging. Symptomatic stiff shoulders can be caused by a variety of underlying conditions, including cervical spine disease, diseases that cause dysfunction of the shoulder joint, and abnormalities in the musculature of the shoulder and neck region. In the psychiatric field, stiff shoulders are often reported in patients with panic disorder, psychosomatic disorders, and depression.^3)^ In addition, stiff shoulders are known to be induced by generalized anxiety and tension in the posterior neck muscles.

(b) History and details of standard treatments that have been implemented to date.

For treatment of shoulder stiffness, treatment of the underlying disease is given priority if it is symptomatic. For essential shoulder stiffness, drug therapy such as trigger point injections, nerve blocks, intervertebral disc injections, intervertebral joint blocks, and nonsteroidal anti-inflammatory analgesics, physical therapy, heat therapy, acupuncture, and phototherapy using low-frequency acupuncture and low power laser, linearly polarized infrared light, etc. are used.

On the other hand, according to the summary of the 1998 National Survey of Living Conditions, more people complaining of stiff shoulders receive treatment at anma, acupuncture, moxibustion, and other treatment centers (54.0%) than at hospitals (23.4%) or clinics (25.7%)^4)^, indicating that not only treatment at medical institutions but also osteopathic clinics and ^4)^, and in many cases, patients receive treatment not only at medical institutions but also at osteopathic clinics and massage parlors, or are treated only by self-care.

(c) Current Standard of Care and Outcomes

As a treatment involving medical treatment, Morimoto et al. reported the results of trigger point injections using a combination of dibucaine hydrochloride and corticosteroids. The report showed a 54% reduction in pain as measured by a visual analog scale.3) Bogduk et al. reported that intervertebral joint block resulted in complete pain relief in 16 of 24 patients.^5)^

5) As for the results of acupuncture and other treatments, Hayashi et al. studied the effects of low-frequency acupuncture on patients with shoulder stiffness for more than one year and reported that treatment improved subjective symptoms of shoulder stiffness and muscle hardness.^6)^ In addition, Kishi et al. ^7)^ However, the evidence for the treatment of stiff shoulders in the field of Oriental medicine is not yet sufficient.

In the field of optical acupuncture and moxibustion, which is the subject of this evaluation, laser-based optical acupuncture and moxibustion have been used for some time. There are also some self-care products available on the market (available for several thousand yen to 20,000 yen via online shopping, etc.). Laser acupuncture has the advantage of being less invasive and requiring less time than conventional acupuncture. On the other hand, the characteristics of lasers (monochromatic, directional, and high coherence) make them unsafe, and strict regulations are imposed on their use. In the past literature on optical acupuncture and moxibustion, parameters such as wavelength, irradiation amount, beam shape, skin thickness of the irradiated area, and age of the research subjects varied, making uniform interpretation difficult,^8)^ and the mechanism of efficacy of optical acupuncture and moxibustion is not well understood.^9)^

(d) Issues, uncertainties, etc. in the current standard treatment that lead to the need for such clinical trial

As mentioned above, stiff shoulders are a very popular ailment, and while treatments such as trigger point injections are effective, on the other hand, people often receive acupuncture, moxibustion, massage, and other treatments without visiting a medical institution. There are a large number of health care devices on the market for stiff shoulders, and the need for such devices is high. Although these health care devices are inexpensive and readily available, scientific verification of their effectiveness is not yet sufficient.

The optical acupuncture device used in this study can generate near-infrared light with a wavelength of 780 nm at an output of 750 mW. Several papers have reported the effects of near-infrared light on the body, including selective inhibition of peripheral nerve excitation conduction, relaxation of vascular smooth muscle, and dilation of arterioles due to sympathetic nerve inhibition.^10,11)^ In animal experiments using rabbits, Komori found that direct irradiation with linearly polarized near-infrared light caused dilation of the auricular arterioles' diameter, blood flow velocity, and blood flow rate. ^12)^ Based on these results, we expect that the same effects will be observed with this acupuncture device. We will use the LED optical acupuncture device in this research project on people who are aware of stiff shoulders and evaluate its safety and effectiveness in relieving symptoms of stiff shoulders, as well as whether it has a favorable effect on physiological tests such as skin temperature, autonomic nervous system, and electromyogram. If the safety and effectiveness of this device are proven, the possibility of evidence-based self-care for stiff shoulders will be found, and its commercialization in the future will improve the health of many people and contribute to the realization of an unaffected, healthy, and long-lived society and a reduction in social security costs.

**2-2.** **Information on medical devices used in the clinical trial**

| Classification | 78 Electrotherapy Apparatus for Home Use |
| --- | --- |
| Generic name | Household infrared therapy apparatus |
| Medical device Class | II |
| Trade name | LED light acupuncture and moxibustion device |
| Purpose or effect | Relief of symptoms of stiff shoulders |
| Usage | Irradiation to acupuncture points |

**LED light acupuncture and moxibustion device**


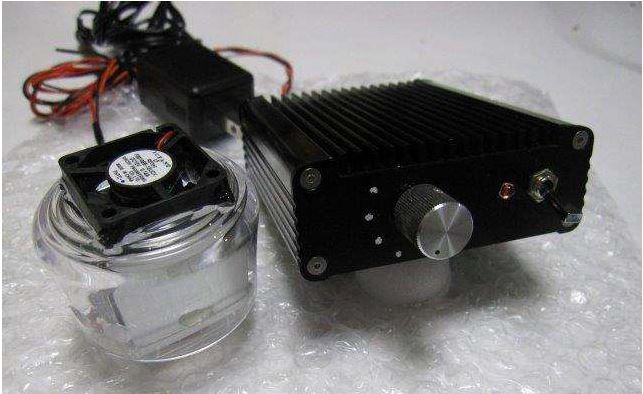


**irradiation device　　　　irradiation section (skin contact section)**

**
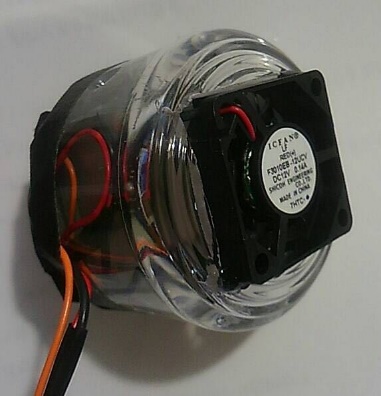

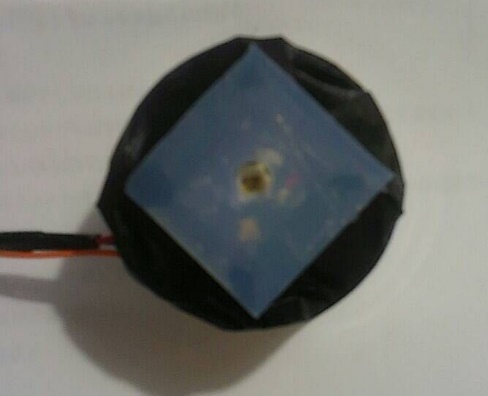
**

silicone rubber

Light Source

LEDs are not monochromatic but broadband light, less directional than lasers, and noncoherent, and are therefore considered safer than lasers. Nevertheless, the 780 nm wavelength is a concern for thermal injury to the retina, low visible light thermal injury to the retina, and infrared radiation injury, so the device is equipped with a safety mechanism that allows the LED to be irradiated only when grounded to the skin.

**3. Matters Related to the Purpose of the Clinical Study**

To verify whether a single treatment using an LED acupuncture device (wavelength 780nm±15nm, output 750mW) on adults who are aware of stiff shoulders causes changes in subjective symptoms (stiffness and pain in the shoulders) evaluated using VAS, pain threshold evaluated using a tenderness meter, autonomic nerve function evaluated using Finapres NOVA and baroreceptor reflex sensitivity, as compared to before the treatment.

**4.** **Matters related to the content of the clinical trial**

**4-1.** **Primary and secondary endpoints**

**4-1-1.** **Primary endpoints**

Subjective symptoms (VAS)

**4-1-2. Secondary endpoints**

Skin temperature, pressure pain threshold, heart rate variability, baroreceptor reflex sensitivity, adverse events

**4-1-3. Exploratory endpoints**

Muscle properties observed on ultrasonography (brightness, muscle contraction, muscle thickness, etc.), changes in surface electromyography (amplitude, frequency, etc.)

**4-2.** **Types of clinical trials, methods, and procedures**

**4-2-1.** **Number of cases**

Planned case enrollment　10 cases
Subjects will be publicly recruited by posting posters, etc.

**4-2-2.** **Trial period**

The expected period of participation of research subjects: approximately two to three days per week

Observation period (enrollment of the first case - completion of data collection for all endpoints): February 1, 2021, to June 30, 2021

Total study period: February 1, 2021, to March 31, 2021

**4-3.** **Inclusion and exclusion criteria for study subjects**

**4-3-1.** **Inclusion criteria**

The following selection criteria will be applied to those who meet all of the following criteria

1. men and women 20 years of age or older

2. who are aware of shoulder stiffness

3. Persons who have given their consent to this study by signing a consent form.

**4-3-2　Exclusion criteria**

Patients who meet at least one of the following exclusion criteria will be excluded.

1. those with diseases of the cervical spine (cervical spondylosis, herniated disc, intervertebral arthritis, cervical sprain, etc.)

2. those who are being treated by a physician for stiff shoulders

3. who habitually receive treatment for stiff shoulders or self-care (e.g., compresses, oral medication, acupuncture, moxibustion, massage)

4. who has skin abnormality from the neck to the back

5. who are deemed inappropriate by the Principal Investigator (or Co-Principal Investigator).

**4-4.** **Methods of enrollment, randomization, blinding of study subjects**

**4-4-1** **Enrollment of Study Subjects**

After obtaining written consent as a research subject, the principal (sub)investigator will ensure that all selection criteria are met and all exclusion criteria are not violated. The principal investigator (or sub-principal investigator) will then assign an identification code, complete the screening roster, and enroll the research subjects.

**4-4-2.** **Randomization Blinding Methods**

Not applicable to this study.

**4-5.** **Overview of Research Chemicals and Equipment**

As stated in 2-2. Information on medical devices used in the clinical trial

**4-6 Research Procedures**

**4-6-1 Research implementation system**

This clinical trial will be conducted as part of the "Obtaining Evidence of Efficacy of LED Optical Acupuncture and Moxibustion" project (project leader: Hiroaki Yokota, Graduate University of the Creation of New Photonics Industries), which was adopted as an industry-academia-government-finance collaboration innovation promotion project, and for which Sasiperere Japan Ltd. applied. The LED acupuncture and moxibustion device to be evaluated is an unapproved medical device, and the clinical trial to be conducted to evaluate its safety and efficacy falls under the category of specified clinical trial and must therefore be conducted at a medical institution. Therefore, the University decided to participate in the project. Neither the principal investigator (Dr. Odagiri) nor the sub-investigator (Dr. Yamauchi) has acupuncture and moxibustion skills or national certification, so the treatment will be performed by Zenba Acupuncture and Moxibustion Clinic's Maeba (acupuncturist) under the supervision of Drs. (former professor at St. Christopher's University, now Health Data House Corporation (Iwate University venture)) and Professor Eda of the Graduate University for the Creation of New Photonics Industries will be in charge of echo, electromyography, and thermography, while Toda of the University will be in charge of baroreceptor reflex sensitivity and autonomic nerve function.

**4-6-2　Protocol Treatment**

　Study Design

Single-arm, open-label, uncontrolled, single-comparison study

Treatment with research equipment

Efficacy and safety assessment

Obtaining written consent

Eligibility Verification and Registration

<Protocol Treatment>

The position of the patient during the protocol is the sitting position, and the following order is used.

[At the time of obtaining consent]

After obtaining consent, a questionnaire (background investigation) will be conducted.

[Before treatment]

1. After resting for 5 minutes, vital signs will be measured.
2. The research subject will fill out the questionnaire (before the procedure).
3. Observe the skin at the treatment site.
4. Measurements will be made in the following order: muscle ultrasonography of both shoulders, surface electromyography, thermography, and pressure pain threshold. At the end of the electromyography, continuous blood pressure monitoring and continuous measurement of heart rate variability are performed.
5. The results of the examination are confirmed, and the treating side is decided.

(Criteria for determining the treating side)

The side with the lower pressure sore meter reading is the treatment side.

[Treatment] 1.

1. irradiate LED light acupuncture needles to the shoulder well (a common acupuncture point for stiff shoulders).

（Irradiate the silicon rubber part of the LED acupuncture needle directly onto the skin of the shoulder well for 30 seconds per irradiation. After irradiation, check the condition of the skin, and continue irradiation after 3 seconds until the skin shows a reddish change to the naked eye from before irradiation. Repeat irradiation up to 6 times.

[Immediately after the treatment]

1. Immediately after the LED acupuncture irradiation, skin observation, thermography, and pressure pain threshold (on the treated side only) should be performed. (After the measurement, cover the treated area with a bath towel.
2. The research subject will fill out the questionnaire (immediately after the treatment).

[15 minutes after the treatment].

1. 15 minutes after the end of irradiation, skin observation, thermography, pressure pain threshold (on the treated side only), muscle ultrasonography, and surface electromyography are measured in this order. The Finapres NOVA will be removed when the tenderness meter measurement is completed.
2. The research subject will fill out a questionnaire (15 minutes after the treatment).
3. After 5 minutes of rest, vital signs will be measured.

[Post-procedure observation: 7 ± 2 days after the procedure.

Skin observation will be performed to evaluate safety.

<Examination and evaluation methods

1. Vital signs: Blood pressure and pulse are measured using a sphygmomanometer.
2. Tension and tenderness meter: Measure the circumference of the shoulder well within 10 mm in diameter twice, and calculate the average value.
3. Thermography:
   1. Measure the body surface temperature of the subject's entire body.
   2. Measure body surface temperature within a range of about 10 cm centered on the area irradiated with LEDs.
4. Continuous blood pressure, heart rate, heart rate variability, and BRS test: Measurements will be made using electrocardiogram electrodes and Finapres NOVA.
5. Surface electromyography: Weight-loading test is performed on the shoulder of the treatment site, and muscle fatigue caused by the loading is evaluated by surface electromyography.
   1. Perform the test in a sitting posture. (2) The subject's arms are placed on a desk at rest, and after a 15-second measurement at rest, a 2-minute measurement of (weight) loading is performed.
   2. The load is applied by holding a 2- to 3-kg weight on the treating side and holding it as follows The upper arm is held horizontally forward and the forearm is flexed 90 degrees vertically upward for 15 seconds, and then the forearm is held in extension for up to 2 minutes with the weight and measured. The arm of the non-treated side should be in a posture to support the upper body so that the muscle load on the trunk is excluded. The loading time is 2 minutes, but may be shortened if the subject does not tolerate the weight load or the maintenance of the posture. (When shortening the loading time, the change in EMG from the state of no muscle fatigue to the state of fatigue is measured, so there will be few problems in data collection.)
6. Measurement of the EMG load is performed on the shoulder on the side to be treated.

The measurement site should be the trapezius muscle (especially from the neck to the shoulder) and the deltoid muscle.

1. Muscle ultrasonography: Measurement is made simultaneously with the above surface EMG examination process.
   1. Echocardiography is performed on the trapezius muscles (especially from the neck to the shoulders) and deltoid muscles at rest and during loading.
   2. Avoid electrode positions for the above muscles when performing echo.
   3. A comparison of echo intensity (luminance, muscle contraction, muscle thickness, etc.) of the muscles under weight lifting is performed.

<Observational Examination/Item>.

After obtaining consent, the background of the research subjects will be investigated.

- Study subject background: age, gender, medical history of cervical spine disease, a survey of medications used
- Vital signs: blood pressure, pulse (heart rate), heart rate variability (LF, HF, LF/HF), baroreflex sensitivity (BRS)
- Clinical findings: skin observation (presence of redness)
- Thermography: skin surface temperature of the whole body and the area to be treated
- Pressure pain threshold: measurement of the circumference of the shoulder well within 10 mm in diameter
- Surface electromyography: measurement of the amplitude and frequency of surface electromyograms generated from the trapezius (especially from the neck to the shoulder) and deltoid muscles over time to determine muscle fatigue associated with loading.
- Muscle ultrasonography: measurement of muscle echo changes (e.g., brightness) over time in the trapezius (especially from the neck to the shoulder) and deltoid muscles during loading.
- Questionnaire: (Background survey) Daily living situation, (Before treatment)
- (Before the treatment) Pain due to stiffness in the shoulders (VAS), evaluation of stiffness in the shoulders (VAS)
- (Immediately after the treatment) Pain due to stiff shoulders (VAS), evaluation of stiffness in the shoulders (VAS), evaluation of sensation during the treatment
- (15 minutes after treatment) Pain due to stiff shoulders (VAS), evaluation of stiffness in the shoulders (VAS), evaluation of sensation during treatment (VAS)
- Adverse event (skin burn)

＜Schedule＞

|  | **VISIT 1** | **VISIT 2** | | | | **VISIT　3** |
| --- | --- | --- | --- | --- | --- | --- |
|  |  | **Day of treatment** | | | | **7 ±2 days after treatment** |
|  |  | Before the treatment | **Treatment** | Immediately after the treatment | 15 minutes after treatment |  |
| Obtaining written consent | **○^＊1^** | |  |  |  |  |
| Background evaluation | **○^*1^** | |  |  |  |  |
| Treatment |  |  | **○** |  |  |  |
| Vital Signs Assessment |  | **○** |  |  | **○** |  |
| Continuous blood pressure heart rate measurement, heart rate variability, BRS test ^*２^ |  |  |  |  |  |  |
| pressure pain threshold |  | **○** |  | **○** | **○** |  |
| Thermography |  | **○** |  | **○** | **○** |  |
| Muscle ultrasonography |  | **○** |  |  | **○** |  |
| Surface electromyography |  | **○** |  |  | **○** |  |
| Skin Observation |  | **○** | **○** | **○** | **○** | **○^*3^** |
| Adverse effect |  |  | **○** | **○** | **○** | **○^*3^** |
| Questionnaire |  | **○** |  | **○** | **○** |  |

*1 Perform either VISIT1 or VISIT2.

*2 If continuous blood pressure/heart rate measurement and BRS test cannot be performed, they will not be performed.

*3 Confirmation by telephone or e-mail is acceptable, but if the principal investigator deems it necessary, the patient must come to the hospital for confirmation.

**4-7-1** **Prohibited concomitant medications and therapies**

Treatment of shoulder stiffness and self-care are prohibited from 3 days before the procedure until the procedure. The application of compresses or ointments to the treated area is prohibited from the end of the post-treatment period until the end of the post-treatment observation, as it will affect skin observation.

**4-7-2** **Concomitant Restricted Drugs and Concomitant Restricted Therapies**

Not set.

**4-8.** **Cease Regulations**

**4-8-1** **Criteria for Discontinuation of Individual Research Subjects**

1. When the research subject requests discontinuation
2. If the subject is found to be ineligible for the study
3. When it is found that the subject does not meet the selection criteria or violates the exclusion criteria after consent is obtained
4. If the patient has been treated for shoulder stiffness and self-care from 3 days before the treatment until the treatment
5. When it is determined that the continuation of the study is difficult due to the occurrence of adverse events
6. Other cases in which the principal investigator determines that the research should be terminated.

**4-8-2** **Rules for Discontinuation of the Entire Study**

The principal investigator will consider discontinuation of this research in the event of an unexpected serious adverse event arising from this research. If the decision to discontinue this research is made by the Principal Investigator, the Principal Investigator will promptly inform the research subjects' physicians and other relevant personnel and will conduct examinations and take appropriate measures to ensure the safety of the research subjects. The principal investigator will also notify the Clinical research review board in writing within 10 days.

**4-9.** **Procedures for Administration of Research Medical Devices.**

The research device used for the research will be loaned free of charge by Saciperere Japan. Appropriate storage and management of the research equipment will be conducted following the instruction manual provided by the loaner (High Luminance LED Onkyu Device, January 2020, TAK SYSTEM INITIATIVE CORPORATION.

**4-10.** **Randomization Method**

As stated in 4-4-2. Randomization Blinding Methods

**4-11.** **Identification of source documents**

Source documents are data and other records obtained through a clinical trial. Electronic data such as case report forms, questionnaires, and acquired images are considered source documents.

**5. Criteria for Selection and Exclusion of Subjects for Clinical trial and Discontinuation of Clinical trial**

5-1. Inclusion Criteria

As stated in 4-3-1. Inclusion criteria

**5-2. Exclusion Criteria**

As stated in 4-3-2. Exclusion criteria

**5-3.** **Criteria for discontinuation of clinical trial**

Stated in 4-8. Cease Regulations

**6.** **Matters relating to the treatment of subjects of clinical trial**

Stated in 4-2. Types of clinical trials, methods, and procedures

**7.** **Evaluation of Efficacy**

**7-1. Efficacy Endpoints**

Primary endpoint: subjective symptoms (VAS)

Secondary endpoints: changes in skin temperature, baroreflex sensitivity (BRS), autonomic nervous system function (heart rate variability)

Exploratory endpoints: muscle properties observed by ultrasonography (luminance, muscle contraction, muscle thickness, etc.), changes in surface electromyography (amplitude, frequency, etc.)

**7-2.** **Evaluation, Recording, and Analysis Methods**

Stated in 9-1. Statistical Analysis Methods

**8. Evaluation of Safety**

**8-1.** **Safety endpoints**

Adverse event (skin burn)

Failure of the treatment device

**8-2.** **Evaluation, recording, and analysis methods**

**8-2-1 Adverse event**

Adverse event means any undesirable or unintended illness or disability, or any manifestation thereof, occurring to Research Subjects or others who used or were used to use the Research Equipment. (A causal relationship to the Research Equipment is not required. (However, for those occurring in persons other than the Research Subjects, it shall be limited to those suspected to be the result of the use of the Research Equipment.)

When an adverse event or a malfunction of the treatment device is observed, the principal investigator (or a sub-investigator) shall immediately observe the patient's general condition appropriately, take appropriate measures, and promptly document the incident in the case report form. The frequency of adverse events and failures will be analyzed at the end of the study.

However, in this study, skin abnormalities at the irradiation site will be addressed, but not limited to, if deemed necessary by the principal investigator.

Among adverse events, events for which a causal relationship with this study cannot be denied shall be treated as "disease, etc.".

**8-2-2 Failure of the treatment device**

A malfunction is defined as a failure in the quality, safety, or performance of research equipment, such as breakage or malfunction, regardless of whether the failure is due to the design, delivery, storage, or use of the equipment.

When an adverse event or failure of the treatment device is observed, the principal investigator (or sub-investigator) should immediately observe the patient's general condition, take appropriate measures, and promptly document the incident in the case report form. At the end of the study, the frequency of adverse events and failures of treatment devices will be analyzed.

**8-3.** **Methods for collecting, recording, and reporting information on diseases and failure of the treatment device**

Procedures for collecting, recording, and reporting information on diseases and failures of treatment devices shall follow the "Standard Operating Procedures for Responding to Disease Outbreaks".

**8-4.** **Response after the occurrence of disease, etc. or malfunction**

Follow the " Standard Operating Procedures for Responding to Disease Outbreaks.

**9.** **Statistical analysis**

**9-1.** **Statistical Analysis Method**

9-1-1 Background characteristics of the population

The background factors of the study population should be described with descriptive statistics (mean, standard deviation, median, quartiles, percentage (%), etc.).

**9-1-2 Analysis of the primary endpoint**

Subjective symptoms (VAS) measured over time before, immediately after, and 15 minutes after the procedure at the treatment site will be analyzed using one-way repeated measure ANOVA or Friedman's test.

**9-1-3** **Analysis of secondary endpoints**

- On the treated side, the pre-, immediate post-, and 15-minute post-treatment tenderness meter and skin temperature measurements over time will be analyzed using one-way repeated measure ANOVA or Friedman's test.
- One-way repeated measure ANOVA or Friedman's test will be used to analyze the pressure pain threshold, skin temperature, and subjective symptoms (VAS) measurements over time before, immediately after, and 15 minutes after the treatment on the non-treatment side.
- Differences between the treated and non-treated sides of the pressure pain threshold, skin temperature readings, and subjective symptoms (VAS) over time before, immediately after, and 15 minutes after the treatment will be analyzed using two-way repeated measure ANOVA or Friedman's test.
- Heart rate variability frequency analysis will be performed to compare changes in the low frequency (LF) range and the power ratio of the low-frequency range to the high-frequency range (LF/HF) from before to 15 minutes after treatment. Average values will be taken before (3 minutes), during (up to 3 minutes), immediately after (5 minutes), and 15 minutes after (5 minutes) the procedure and analyzed using One-way repeated measures ANOVA or Friedman's test.

**9-1-4** **Exploratory analysis**

- Compare the changes (amplitude, frequency) in surface electromyography due to weight loading before and after 15 minutes of treatment
- Muscle properties (luminance, muscle contraction, muscle thickness, etc.) observed by muscle ultrasonography will be compared before and 15 minutes after the treatment.
- Comparison of baroreceptor reflex sensitivity before, immediately after, and 15 minutes after the procedure

**9-2.** **Interim Analysis**

Not performed

**9-3.** **Establishment of the number of cases**

This was a feasibility study, and the number of patients was not set on a statistical basis. The minimum number of patients who could feasibly be evaluated for the endpoints was approximately 5-7, and 10 patients were chosen to account for dropouts.

**9-4.** **Significance Levels**

The significance level is p<0.05 for all tests.

**9-5.** **Discontinuation Criteria**

As stated in 4.8 cease Regulations

**9-6.** **Data Handling**

(Analysis Population)

Efficacy analyses will be performed on the largest analysis population (FAS). If necessary, a sensitivity analysis of efficacy will be conducted on a population that conforms to the research protocol (PPS).

(Safety analysis)

All study subjects (SP) will be included in the study, and subjects will be grouped according to whether or not they received the intervention. Consideration of the treatment of individual study subjects and determination of the attributable analysis population will be made before data fixation.

Reasons for Exclusion FAS PPS SP

|  | FAS | PPS | SP |
| --- | --- | --- | --- |
| Study subjects who did not receive any treatment | Exclusion | Exclusion | Exclusion |
| Study subjects who did not receive any investigation of efficacy endpoints | Exclusion | Exclusion | Included |
| Research subjects who violated the selection and exclusion criteria | Included | Exclusion | Included |
| Research subjects who deviated from the research protocol | Included | Exclusion | Included |

**9-7.** **Procedure for changing the analysis method**

The principal investigator shall revise the research protocol and explain any changes from the original statistical analysis plan in the clinical trial summary report.

**9-8.** **Selection of Analysis Subjects**

As stated in Data Handling

**10.** **Access to source documents, etc. (including the contents of contracts entered into under Article 32 of the Clinical Trials Act).**

The head of the institution and the principal investigator will provide direct access to all clinical trial-related records, including original documents, during monitoring, audits, and investigations by the Clinical research review board and regulatory authorities related to the research in question.

**11.** **Quality Control and Quality Assurance**

The Principal Investigator shall control and assure the quality of the clinical trial by having the monitors monitor the progress of this clinical trial to confirm that this clinical trial is being conducted appropriately following the research protocol and the Clinical trials Act. The principal investigator will prepare a separate monitoring protocol describing the items to be checked and the responsibilities of the parties involved, etc., and the monitors will conduct monitoring following the monitoring protocol.

No audits will be conducted.

**12.** **Ethical Considerations**

**12-1．Approval by a certified research review board**

The appropriateness of conducting this research will be reviewed and approved by the clinical research review board of the Hamamatsu University School of Medicine and approved by the administrator of the implementing medical institution.

**12-2.** **Benefits accruing to subjects of clinical trial**

Participation in this study is expected to provide relief from shoulder stiffness in the treated area. In addition, information derived from blood perfusion, contractile and relaxing functions in the shoulder muscles will be obtained and may be useful for future improvement of shoulder stiffness.

**12-3.** **Burden and foreseeable disadvantages to subjects of clinical trial**

Each study will require approximately one hour for preparation, treatment, and measurement.

The anticipated adverse event for the LED light acupuncture device is considered to be a burn of the skin at the treatment site.

**13.** **The handling and preservation of records (including data).**

**13-1.** **Provision of records to third parties**

Records (including data) obtained in this study will be anonymized at the implementing medical institution so that they cannot be identified at first glance, and will be sent to the following. When the information is provided, it will be managed responsibly by the principal investigator, and no correspondence will be provided to identify the information and individual research subjects. The information provided will be appropriately stored by the person in charge of the relevant medical institution as described below.

The information to be transferred, the name of the research institution, and the responsible person are as follows.

- Samples and records (including data) to be transferred

Records (including data): Records (including data) obtained in this study (excluding correspondence table)

- Name and responsible person of the research institute to which the transfer is to be made
  - The Graduate School for the Creation of New Photonics Industries; Hiroaki Yokota
  - Isehara Clinical trial center, Zenba-acupuncture and moxibustion clinic; Kazuyoshi Zenba
  - Kenkou Data House Inc.; Hiroaki Okawai

**13-2.** **Methods of retaining and disposing of samples and information**

Paper information about the research will be stored in a locked archive at the Center for Clinical research. Electronic data will be stored on a hard disk managed by the principal investigator at the Center for Clinical research. At the end of the research, the electronic data will be stored on CD-R or DVD in a locked archive at the Center for Clinical research, together with the paper-based information about the research. Information about the research will be destroyed after a specified period (10 years from the date of the final publication of the results of the research).

**13-3.** **Handling of research results related to research subjects**

1. if the individual subject of the research wishes the information to be disclosed, the results will be disclosed.
2. If the research subject does not wish the information to be disclosed, the results will not be disclosed.
3. If a person other than the research subject wishes the information to be disclosed, the results will not be disclosed in principle.

**14.** **The payment of money and compensation for the conduct of the clinical trial**

**14-1.** **Payment of monies related to the conduct of the clinical trial**

The honorarium to the research subjects for participating in this study will be 2000 yen worth of electronic money.

**14-2. Insurance and Compensation**

This clinical trial is covered by clinical trial insurance. If health damage occurs to research subjects as a result of the implementation of the research, the principal investigators and sub-investigators will provide adequate medical treatment and other appropriate measures and will endeavor to investigate the cause of the damage. Medical expenses for such treatment will be covered by health insurance, and the research subjects will pay for any out-of-pocket expenses.

In addition, compensation will be paid by the clinical trial insurance for death or disability of classes 1 to 14 under the Adverse Effects Relief System for Drugs.

**15.** **The publication of information on clinical trial**

Before starting a study, the details of the research plan shall be recorded in the jRCT (Japan Registry of Clinical Trials), a database maintained by the Ministry of Health, Labour and Welfare (MHLW), and made public.

**16.** **Duration of Clinical Trial**

As stated in 4-2-2. Trial period

**17.** **Explanation of subjects of clinical trial and their consent (including the form to be used for these)**

The principal investigator shall prepare the explanation and consent documents used to obtain consent from clinical trial subjects following Article 46 of the Enforcement Regulations of the Clinical Trials Act and related guidance, and shall revise these documents when necessary. The explanation and consent documents should be approved in advance by the clinical research review board.

Before obtaining consent from a research subject, the principal investigator or a research assistant physician must give the research subject the opportunity to ask questions and sufficient time to decide whether or not to participate in the research. At that time, the principal investigator, research associate, or research collaborator as an assistant explainer must answer all questions to the satisfaction of the research subjects. After confirming that the research subject understands the content of the research, the research subject's free and voluntary consent to participate in this research will be obtained in writing.

The consent document should be signed and dated by the principal investigator or research associate and the research subject. If a research collaborator assists in providing explanations, he/she should also sign and date the document.

The principal investigator or sub-investigator should provide the research subject with a copy of the signed and dated consent form and written explanation before the subject participates in the research, and the original consent document should be stored appropriately following the regulations of the relevant medical institution.

In addition, points to note regarding obtaining consent and revisions to the explanation and consent documents are indicated below.

<Considerations for Obtaining Consent

(1) The Principal Investigator, Research assigning Physician, and Research Collaborators shall not coerce or exert undue influence on the research subjects concerning their participation or continued participation in the research.

(ii) The explanatory documents shall use plain, non-technical language that is understandable to research subjects to the greatest extent possible.

(iii) Explanation and consent for research subjects who are unable to read the explanatory document should be fully explained in the presence of an impartial observer, and consent should be obtained of the subject's own free will. The witness should also sign and date the consent document. An impartial witness is a person who is independent of the conduct of the research and is not unduly influenced by those involved in the research, and must not be the principal investigator, a research physician, or a research collaborator.

<Revision of Explanatory and Consent Documents

(1) When new important information affecting the research subject's willingness to consent is obtained, the principal investigator or sub-investigator will immediately convey such information to the research subject, confirm the subject's willingness to continue participating in the research, and record the information in writing. The principal investigator will also promptly determine whether or not to revise the explanation and consent documents based on such information.

(ii) If the principal investigator finds it necessary to revise the explanation and consent documents, he/she should do so promptly and obtain another approval from the clinical research review board.

(iii) The principal investigator or sub-investigator should explain to the research subjects already participating in the research using the explanation and consent documents that have been approved anew by the clinical research review board and obtain written consent from the research subjects for their continued participation in the research, of their own free will.

The principal investigator or sub-investigator should provide the research subject with a copy of the newly signed and dated consent document and the explanation document, and appropriately store the original consent document following the regulations of the relevant medical institution.

＜Secondary Use of Samples and Information

　When planning new research using samples and information collected in this study, a new research plan should be prepared, approved by an appropriate ethics committee, etc., and in compliance with laws, regulations, and other rules.

**18.** **Other matters necessary for the proper conduct of the clinical trial in addition to those listed above.**

**18-1.** **Rules and Regulations to be complied with**

All investigators involved in this research will conduct this research in compliance with the "Declaration of Helsinki" and the "Clinical Trials Act and the Enforcement Regulations of the Clinical Trials Act".

**18-2.** **Conflict of Interest Management Plan**

Before the implementation of this study, the Principal Investigator shall survey all responsible (co-Principal Investigators) at all of the medical institutions involved to determine whether or not they have any conflicts of interest with related companies, and shall prepare a Conflict of Interest Management Standard and a Conflict of Interest Management Plan for all persons reporting conflicts of interest in this study. Along with these materials, conflicts of interest related to this research will be clearly stated in the research protocol, submitted to the Clinical research review board for review and approval, and conflicts of interest will be continuously checked and managed appropriately throughout the implementation period of this research. The details will be described in the explanatory document, and consent for participation in this research will be obtained after explanation to the research subjects.

This research will be conducted using funds from t the Access Center for Innovation Solutions, Actions and Professionals (A-SAP) from the Photon Valley Center, the Hamamatsu Agency for Innovation. Phase 6 project, "Obtaining Evidence of the Efficacy of LED Optical Acupuncture. There is no funding from Saciperere Japan for this research. There are no "possible conflicts of interest" in the design, conduct, or reporting of this study that would affect the results of the study or the interpretation of the results. The conduct of the research will not prejudice the rights or interests of the research subjects.

There is no provision of research funds or other involvement in the clinical trial conducted by the principal investigator by a pharmaceutical manufacturer or distributor (pharmaceutical manufacturer or distributor or its special related party).

Contributions, manuscript writing, remuneration for lectures or other work, or other involvement by a manufacturer or distributor of pharmaceuticals, etc. that manufactures or sells pharmaceuticals, etc. used in a said clinical trial to persons engaged in a clinical trial conducted by the said principal investigator and persons listed in the research protocol who will benefit from the conduct of the said clinical trial.

**18-3.** **Reasons why explanation and consent are not required**

Not applicable

**18-4.** **Attribution of Results**

The ownership of the results, data, and intellectual property rights obtained from this research will be discussed separately with Hamamatsu University School of Medicine and The Graduate School for the Creation of New Photonics Industries.

**18-5. Withdrawal of Consent**

If a research subject withdraws or refuses to give consent under any of the circumstances described below, the principal investigator and the sub-investigator will take measures following the details of the withdrawal or refusal without delay, and will explain to the research subject to that effect. However, this shall not apply in cases where it is difficult to take such measures and after obtaining the opinion of the Clinical research review board on not taking such measures. In such a case, the principal investigator and the sub-investigator will explain to the research subjects that measures will not be taken following the content of the withdrawal or refusal and the reasons for not taking such measures and will endeavor to obtain their understanding.

1. Withdrawal of all or part of the consent given for the research to be conducted or continued
2. Refusal, in whole or in part, to allow the research to be conducted or continued, based on information that has been communicated or disclosed about the research
3. Refusal, in whole or in part, to allow the research to be conducted or continued in the handling of the research under circumstances of immediate and obvious life-threatening risk to the research subjects.

**18-6** **Compliance with the trial protocol**

The Principal Investigator(s) will conduct this clinical trial in compliance with the research protocol, except to avoid immediate danger to the research subjects or for other medically unavoidable reasons.

**18-7** **Changes or revisions to the trial protocol**

When the principal investigator changes or revises the research protocol or the explanation document, consent form, or consent withdrawal form, he/she will report the details of the changes or revisions to the clinical research review board in advance and obtain approval.

**18-8 Nonconformity Management**

**18-8-1** **Nonconformity**

When the Principal Investigator becomes aware that this clinical trial is not in conformity with the Clinical Trials Act (including the Enforcement Regulations and related notifications) or the research protocol ("nonconformity"), he/she will promptly report it to the Administrator of the site of implementation. When the Principal Investigator becomes aware of a Non-Conformity, he/she shall promptly report it to the Responsible Investigator.

**18-8-2** **Critical Nonconformity**

A critical nonconformity affects the human rights or safety of the subjects of the clinical trial, the progress of the research, or the reliability of the results. However, it does not include cases in which the research protocol was not followed to avoid immediate danger to the research subjects or for other unavoidable medical reasons. The following are examples in this study.

In the event of any of these potentially serious non-compliances, the principal investigator will report the situation to the Clinical research review board as soon as the situation is ascertained. In addition, measures to prevent recurrence should be taken and communicated to the investigators to ensure that recurrence does not occur again.

1. Critical non-compliance with eligibility criteria

- The investigator knowingly (falsely) enrolled a patient who did not meet the eligibility criteria.
- Enrolled and conducted research without necessary informed consent.
- ∙Unable to identify source documents to determine eligibility (including missing consent forms).

1. Violation of protocol

Violations that have an impact on an increased risk to research subjects or that affect the reliability of research results

- Serious violation of eligibility/exclusion criteria
- Violation of discontinuation criteria that jeopardize the safety of research subjects
- Intentional or systematic noncompliance with protocol provisions

1. Other serious noncompliance

- Implementation of research before approval by the Clinical research review board or before approval by the President (Administrator of the implementing institution).
- Continuation of research without providing information could have affected the physician continuing the research.
- Research misconduct (e.g., fabrication or falsification of data)
- Leakage of personal information or infringement of human rights could have a serious impact on research subjects.
- State of being funded without concluding a contract with a manufacturer or distributor of research equipment.
- State in which falsehoods, etc. are found in the conflict of interest (COI) management documents.

**18-9** **Periodic Reporting**

The principal investigator shall, in principle, report to the Administrator of the institution on the status of implementation of this research every year starting from the day the implementation plan is disclosed, and then report to the Clinical research review board.

Regarding the periodic report, the status of implementation of this research shall be reported to the Minister of Health, Labour, and Welfare within one month from the date on which the Clinical research review board has expressed its opinion on the periodic report.

**19.** **References**
１）Morimoto M、Katakori no rinshou：Tekisetsuna　chiryou to sindan notameni、Kinnkidaiisi　35、151-156、2019 (iin Japasene)

２）Ministry of Health, Labour and Welfare, 2019 National Survey, https://www.mhlw.go.jp/toukei/saikin/hw/k-tyosa/k-tyosa19/index.html

３）Shimizu Y, et al、Shinsinsho　sinkeisho　no　tkatakori　-　katakorinochiryouga　sinsinsho　sinkeisho　no　ninnchikoudoumennnimotarasukoukanitiote. Shinsinigaku.2001,41,645-696 (in Japanese)

４）Ministry of Health, Labour and Welfare, Summary of the 1998 National Survey of Living Standards, [https://www.mhlw.go.jp/www1/toukei/h10-ktyosa/index_8.html](about:blank) (in Japanese)

５）Bogduk N et al., The cervical zygapo-physicaljoints as a source of neck pain, Spine, 1990, 13, 610-617

６）Hayashi K,et al. Katajoubu no katakori ni taisuru teishuuhasintuudennryouhou no kouka- chiryohindo wo kouryishita kentou, Toyoigakukei butsuri ryohogakkaishi ,2016,41,73-79 (in Japanese)

７）Kishi K,et al. Kenseiketu ni taisusukyusigeki ga oyobosu kinkoukertu to ketuatu to sinpakusuuhenkanituite Touyouryouhougakkoukyoukaigakkais,, 2018,42,81-83 (in Japanese)
８） Whittaker, P. Laser acupuncture: past, present, and future. Lasers Med Sci 2004, 19, 69-80

９） Chon, T. Y. et al., Laser Acupuncture: A Concise Review. Med Acupunct 2019, 31, 164-168

10) Hosokawa T,Teihannoureberureza pain clinic,2005,26,662-670 (in Japanese)

11) Saeki S et al,Teihannoureberureza ni yoruitamichiryo painclinic,2005,26,791-801.(in Japanese)
12) Komori M,Teishuturyokurezaoyobi chokusenhenkoukinsekigaisenchokusetushosha no　hifubishojunkan nitaisurukouka, Nihonrezasigakkaisi, 2008,19,26-29 (in Japanese)
